# Case report: Abomasal ulcer secondary to congenital pyloric and duodenal stenosis in a 3-day-old heifer

**DOI:** 10.3389/fvets.2023.1235110

**Published:** 2023-10-11

**Authors:** Fatima Zahra Laabouri, Chelsea Folmar, Vicente Avila Reyes, Erin Beasley, Clare Ryan, Corrie Brown

**Affiliations:** ^1^Department of Pathology, College of Veterinary Medicine, University of Georgia, Athens, GA, United States; ^2^Department of Medicine, Surgery and Reproduction, Hassan II Institute of Agronomy and Veterinary Medicine, Rabat, Morocco; ^3^Department of Large Animal Medicine, College of Veterinary Medicine, University of Georgia, Athens, GA, United States

**Keywords:** abomasal ulcer, congenital abnormality, duodenum, peritonitis, heifer

## Abstract

Abomasal ulcers, an economic concern for all calf-raising farms, are usually silent until perforation occurs, at which time management is complicated and often unrewarding. This case study describes perforating ulcer in a 3-day-old Brahman heifer, occurring secondary to a congenital narrowing of the pylorus and proximal duodenum and leading to marked abomasal distention, leakage, and eventual peritonitis and sepsis.

## Introduction

Abomasal ulcers are lesions in the abomasum that penetrate the entire thickness of the mucosa and may extend through the submucosa and muscularis externa and reach the serosa. These may be single or multiple lesions (1, 2), and their size may range from a few millimeters to several centimeters (3). Abomasal ulcers in calves are classified into four types. Type 1 ulcers are non-perforating ulcers, with minimal intraluminal hemorrhage and local wall thickening and serositis, type 2 ulcers are non-perforating ulcers with severe intraluminal hemorrhage, type 3 ulcers are perforating ulcers with local, confined peritonitis, and type 4 ulcers are perforating ulcers with a generalized peritonitis after ingesta spills into the abdominal cavity (1, 4, 5). Ulcer management is complicated in calves and the causes are multifactorial.

Ante-mortem diagnosis is often challenging. Clinical signs may be absent, subtle, or severe, ranging from general signs of gastrointestinal discomfort such as non-specific abdominal pain, dehydration, anorexia, and hypomotility of the rumen (4, 6) to heavy bleeding or perforation of the abomasum, with signs of anemia, peritonitis, and death (7, 8). In dairy calves, abomasal ulcers are often inapparent and commonly identified in animals dying from other problems or at slaughter (1). They can represent 22% of losses in veal calves (9). At the time of slaughter, the prevalence of abomasal lesions in cows has been reported in the range of 11%−49% (6, 10, 11).

The factors contributing to the development of the abomasal ulcers are numerous, with stress factors topping the list (9, 12). Diet is also thought to play an important role, with many ulcers occurring at the transition from preruminant to ruminant digestion, i.e., at weaning (13). Other factors noted in the literature include low feeding frequency, feeding of abrasive agents, ingestion of stones, mineral deficiencies, notably copper, and administration of NSAIDs (12–16). Infections associated with some fungi and bacteria have also been associated with abomasal ulcers but likely invade the ulceration (17, 18). The case presented here was very unusual and occurred secondary to congenital pyloric and duodenal stenosis.

## Case description

A 3-day-old Brahman heifer was presented for necropsy after written informed consent was obtained from the owner. History included failure to thrive and suspected sepsis that progressed to signs of abdominal distension and discomfort. The calf was treated at the University of Georgia Veterinary Teaching Hospital with a variety of therapeutic modalities, most notably intravenous fluid therapy, including dextrose supplementation, antibiotics, and non-steroidal anti-inflammatory drugs. Abomasal decompression was performed as well. However, in the absence of significant clinical improvement, the calf was humanely euthanized.

External examination at necropsy revealed a state of dehydration, with sunken eyes and tacky subcutaneous tissues. The umbilicus was dry and unremarkable. A distinct red line was present at the gingiva adjacent to the teeth (“toxic line”), indicating probable sepsis, as suspected clinically.

**Figure 1:**
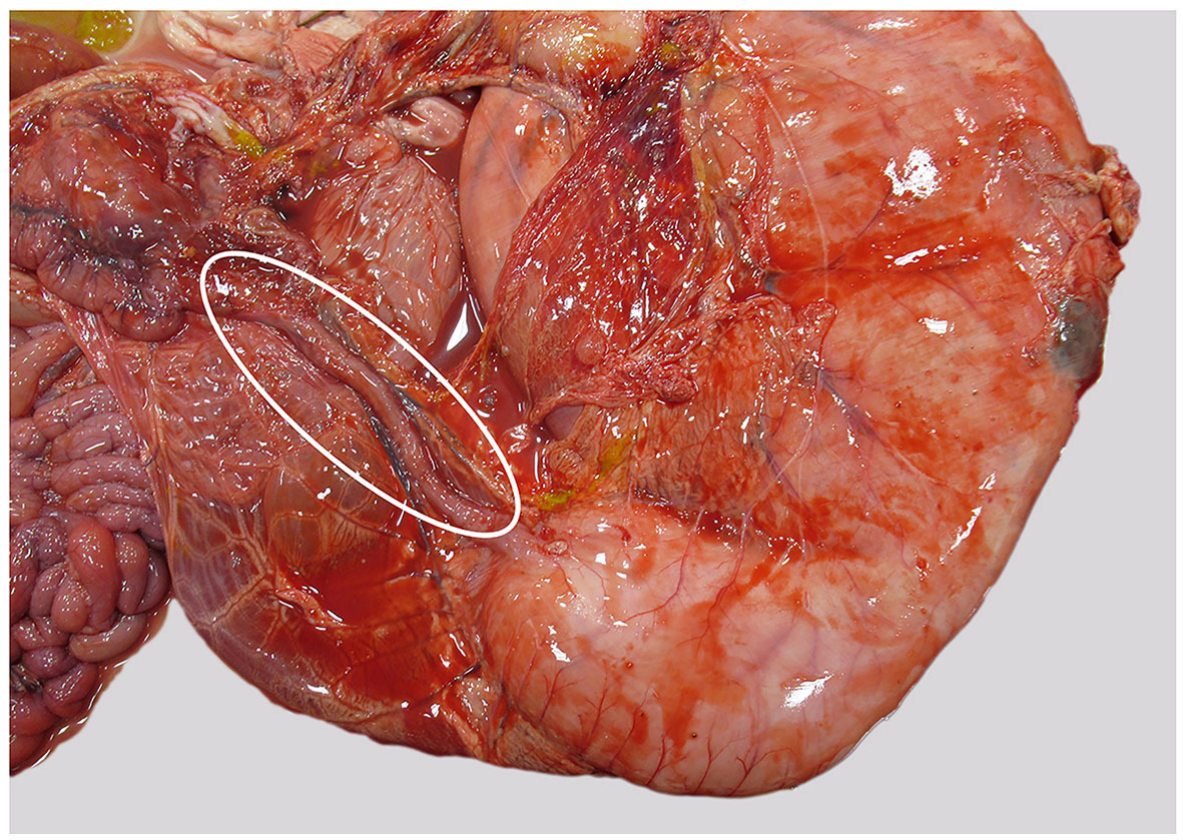
Abomasum and duodenum, as seen at necropsy. Duodenum is markedly narrowed (within oval), abomasum is distended, and fibrin is present along the greater curvature.

Internally, the most remarkable gross finding was a massively expanded, milk-filled abomasum. The expected size of the abomasum was 3–4X, and distinct plaques of fibrin were present at one area of the serosa along the greater curvature. Small amounts of fibrin were seen elsewhere in the abdominal cavity. The pyloric opening was markedly small (8 mm in diameter), and the proximal duodenum was similarly constricted, with a markedly decreased diameter compared with normal, for the proximal 20 cm (see Figure 1). Although data on the normal diameter of the pylorus in cattle could not be found, the figure for normal diameter in domestic cats is 9 mm (19). Milk was also present in the rumen. Multiple large joints were opened. A small amount of fibrin was evident at the occipito-atlanto articulation and in the limbs; there was marked peri-articular redness and edema and occasionally excessive and slightly turbid joint fluid. Aerobic culture from a joint fluid swab yielded no significant growth.

**Figure 2:**
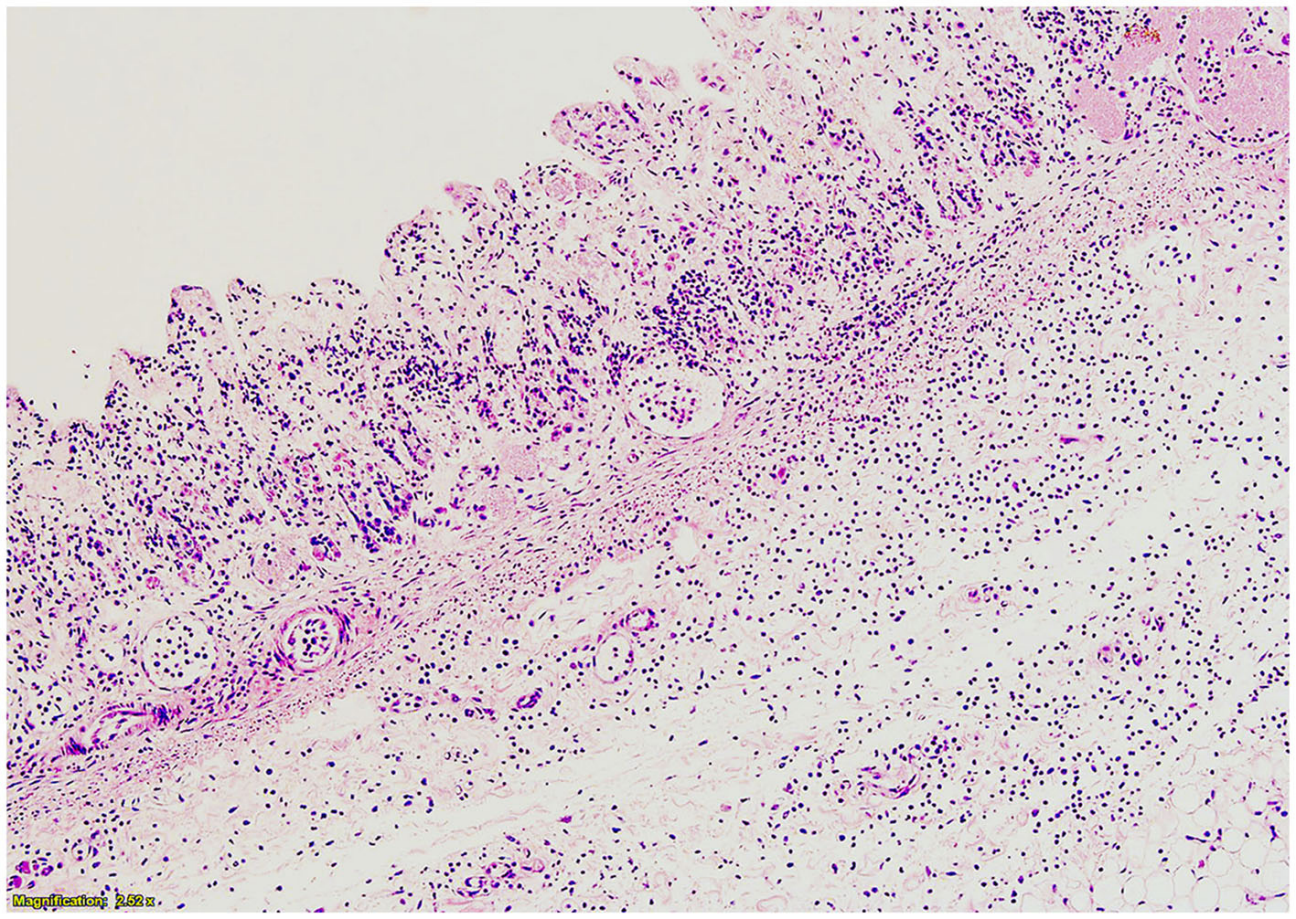
Histopathology of abomasum at greater curvature. Tunica mucosa is diminished, and neutrophils and edema are present throughout the submucosa.

Histologically, the abomasum in a focal area showed a marked diminution of the tunica mucosa and scattered inflammatory cells, predominantly neutrophils, present throughout the submucosa and muscularis, both of which were markedly stretched and with abundant edema (see Figure 2). Subjacent to this, there were fibrin plaques with embedded neutrophils adherent to the serosa and also extending out beyond the serosa. Diffuse congestion and edema were present throughout the lungs. Scattered small aggregates of inflammatory cells, predominantly mononuclear, were present within some parts of the brain, especially in the midbrain. The tissue from the umbilicus was expanded by edema with scattered inflammatory cells, predominantly lymphocytes, and a few distinct clusters of neutrophils (presumed normal postpartum inflammation). Other examined tissues, including the intestines, kidney, bladder, thyroid/parathyroid, adrenal, liver, heart, and spleen, were all histologically unremarkable.

The cause of this calf's clinical problem was presumed sepsis. The marked narrowing of pylorus and duodenum created difficulty in passage of milk, undoubtedly also contributing to the animal's decline. Minimal ingesta was able to pass through the small opening, which resulted in milk accumulation and marked distension of the abomasum, with sufficient compromise of the wall to allow bacteria or milk molecules to enter the peritoneal space. Fibrin plaques directly over the area of the greater curvature which was where the most mucosal compromise was noted are strongly suggestive that leakage was occurring. The lack of significant aerobic bacterial growth from the joint swab is likely because the calf received antibiotics prior to death, resulting in inhibited growth *in vitro*.

In general, abomasal ulceration is a common cause of death in suckling calves aged 4–8 weeks (15, 20, 21). This case is unusual in the very young age of the calf. This heifer was only 3 days old when it was euthanized, after 2 days of intensive hospital therapy. Diagnosis of perforating abomasal ulcer in the calf of <1 week old is striking. Other cases of abomasal ulceration in such young calves are single reports and attributed to severe *in utero* stress (22) or *in utero* fungal infection (18). To the best of our knowledge, this is the first report of abomasal ulceration secondary to congenital pyloric and duodenal stenosis.

## Data availability statement

The original contributions presented in the study are included in the article/supplementary material, further inquiries can be directed to the corresponding author.

## Ethics statement

Written informed consent was obtained from the owners for the participation of their animals in this study. Written informed consent was obtained from the participant/patient(s) for the publication of this case report.

## Author contributions

All authors listed have made a substantial, direct, and intellectual contribution to the work and approved it for publication.
